# The precision medicine process for treating rare disease using the artificial intelligence tool mediKanren

**DOI:** 10.3389/frai.2022.910216

**Published:** 2022-09-30

**Authors:** Aleksandra Foksinska, Camerron M. Crowder, Andrew B. Crouse, Jeff Henrikson, William E. Byrd, Gregory Rosenblatt, Michael J. Patton, Kaiwen He, Thi K. Tran-Nguyen, Marissa Zheng, Stephen A. Ramsey, Nada Amin, John Osborne, UAB Precision Medicine Institute, Stephen Barnes, Matthew Might

**Affiliations:** ^1^The Hugh Kaul Precision Medicine Institute, University of Alabama at Birmingham, Birmingham, AL, United States; ^2^Department of Neurobiology, University of Alabama at Birmingham, Birmingham, AL, United States; ^3^Groovescale, Seattle, WA, United States; ^4^Department of Molecular and Cellular Biology, Harvard College, Cambridge, MA, United States; ^5^School of Electrical Engineering and Computer Science, Oregon State University, Corvallis, OR, United States; ^6^John A. Paulson School of Engineering and Applied Sciences, Harvard University, Cambridge, MA, United States; ^7^Department of Medicine, Informatics Institute, University of Alabama at Birmingham, Birmingham, AL, United States

**Keywords:** rare disease, precision medicine, drug repurposing, artificial intelligence, biomedical reasoning

## Abstract

There are over 6,000 different rare diseases estimated to impact 300 million people worldwide. As genetic testing becomes more common practice in the clinical setting, the number of rare disease diagnoses will continue to increase, resulting in the need for novel treatment options. Identifying treatments for these disorders is challenging due to a limited understanding of disease mechanisms, small cohort sizes, interindividual symptom variability, and little commercial incentive to develop new treatments. A promising avenue for treatment is drug repurposing, where FDA-approved drugs are repositioned as novel treatments. However, linking disease mechanisms to drug action can be extraordinarily difficult and requires a depth of knowledge across multiple fields, which is complicated by the rapid pace of biomedical knowledge discovery. To address these challenges, The Hugh Kaul Precision Medicine Institute developed an artificial intelligence tool, mediKanren, that leverages the mechanistic insight of genetic disorders to identify therapeutic options. Using knowledge graphs, mediKanren enables an efficient way to link all relevant literature and databases. This tool has allowed for a scalable process that has been used to help over 500 rare disease families. Here, we provide a description of our process, the advantages of mediKanren, and its impact on rare disease patients.

## Introduction

It is a challenge in translational research to efficiently bridge discoveries in biomedical research with clinical application. To address this issue, the National Center for Advancing Translational Sciences (NCATS) launched the Biomedical Data Translator program to accelerate translational medical research for furthering research discoveries and clinical implementation (Biomedical Data Translator Consortium, 2019). As part of this program, The Hugh Kaul Precision Medicine Institute (PMI) at the University of Alabama at Birmingham (UAB) Heersink School of Medicine has developed the artificial intelligence (AI) reasoning tool, mediKanren. mediKanren uses knowledge graphs (KGs) produced by teams in the NCATS Biomedical Data Translator program to provide a standardized way of describing the relationship between biomedical concepts (e.g., diseases, symptoms, drugs, proteins, and genes) (Biomedical Data Translator Consortium, 2019). KGs are organized into “triples” which consist of an edge connecting two concepts by a predicate (e.g., “gene A—regulates—gene B” or “gene B—is related to—disease Y”). Unlike typical literature searches, mediKanren triples with related concepts can be linked together, thus providing knowledge that would have otherwise required higher order thinking to establish these connections from multiple sources. Additionally, mediKanren returns this knowledge in a format that allows users to recognize both well-established concept connections and those that may not be well-known. Both types of connections enable the identification of novel therapeutics for rare and complex diseases.

There is a dire need for identifying treatment options for rare and complex diseases. Over 6,000 unique rare diseases have been identified, and it is estimated that over 300 million people worldwide are affected by a rare disease (Nguengang Wakap et al., 2020). Of these rare diseases, approximately 95% do not have an FDA-approved drug treatment and 80% are genetic in origin^1^. Similarly, complex diseases include disorders that involve multiple genes, have gene-environment interactions, and/or encompass related and unrelated symptoms that may not respond to standard treatments. As the clinical application of genome sequencing continues to expand, there is a greater chance for families and individuals to receive an accurate diagnosis. However, this increasing number of rare diagnoses puts pressure on the scientific research community to characterize these diseases and on clinicians to match patients with treatment options (Tabor and Goldenberg, 2018).

PMI has created a research consultation service that analyzes patient cases and uses mediKanren to aid in treatment identification and in disease characterization. In addition to mediKanren, the PMI research consultation program consists of a team of researchers, clinicians, and undergraduate analysts, of various disciplines, including neurobiology, precision oncology, genomic medicine, pharmacology/toxicology, and computer science. The research consultation service consists of (i) analyses of health-related data, including genetic data, of an individual's diagnosed medical conditions or the symptoms of their undiagnosed conditions and (ii) a consultation process that provides insight into the knowledge returned by mediKanren, either in the form of a physician report that outlines mediKanren-identified therapeutic options or a research report that outlines strategies for furthering disease characterization. It is important to note that the consultation process works in partnership with, but does not replace the role of, the participant's physician. In this article, we explain how the PMI research consultation process works, how mediKanren is used in rare and complex diseases, as well as demonstrate this process in selected cases where a therapeutic option was identified.

## Materials and methods

### The artificial intelligence tool mediKanren

mediKanren is a reasoning engine developed under the NIH NCATS Biomedical Data Translator program (Biomedical Data Translator Consortium, 2019). It is based on miniKanren, a family of embedded logic programming languages especially suited for programming with pure relations and constraints. A medical knowledge graph is transformed into a database format that mediKanren then imports, exposing the graph to the programmer as a set of relations. These relations may be used like any other miniKanren relation, efficiently supporting queries in any direction by making use of database indexes. Typical relations built from a knowledge graph describe concepts (e.g., drugs, genes, diseases) and relationships between these concepts.

Each relationship between concepts is a claim (e.g., Drug X treats Disease Y) expressed as a graph edge in subject-verb-object form, where the subject and object are the concepts, and the verb is a predicate (e.g., “negatively regulates”) coming from a standardized ontology. A graph edge comes with annotations that describe features such as information provenance and various forms of evidence, such as publications, supporting the claim expressed by the edge.

Each concept is referenced by a CURIE^2^ (Compact Uniform Resource Identifier) and comes with annotations describing features such as a category (e.g., “gene”) coming from a standardized ontology, a human-readable name, and information provenance. In many cases, two or more concepts are synonyms, using different CURIEs to name the same underlying concept. In other cases, two or more concepts are not exactly equivalent but should be treated as synonyms for a particular reasoning use case. For these reasons, mediKanren supports optional concept normalization, allowing any concept to be substituted for one of its situationally declared synonyms. This enables the discovery of paths through a graph that would otherwise have been disconnected due to edges that mention different CURIEs for the same underlying concept. Because a CURIE can be an obscure way to refer to a concept, mediKanren also supports full-text search over the human-readable English names of concepts. This allows a user, in this case the student analysts, to type in a more familiar name, or even just part of a name, and discover one or more corresponding CURIEs.

The source code for mediKanren is covered under the MIT Free Software license, and is available at https://github.com/webyrd/mediKanren; however, mediKanren requires data contained in knowledge graphs, which require a separate download from a third-party. Contact the authors for help evaluating new uses of mediKanren.

### Third-party knowledge graphs

mediKanren consumes structured data, as opposed to free text. Within NCATS Translator, teams dedicated to the construction of structured data are called “Knowledge Providers” (KPs), and the data sets they produce are known as “knowledge graphs” (KGs) (Biomedical Data Translator Consortium, 2019). Knowledge graphs are derived from various sources, including scientific free text, semi-structured data from electronic health records, and pre-curated biomedical databases. Each KG team is responsible for making their knowledge conform to a common ontology, known as a “Biolink” (Biomedical Data Translator Consortium, 2019). One central team in NCATS Translator is responsible for equating identities between knowledge graphs, creating a special KG called the “Node Normalizer”.

### PMI consultation service data storage and management of cases

After being consented to the research and agreeing to participate, each participant's research consultation process is tracked as an individual PMI case. PMI uses UAB's HIPAA-compliant ShareFile^3^ system to store participants' consultation-related information. After agreeing to participate in the PMI research consultation service, participants themselves or their physicians upload health information through the file-drop feature of UAB ShareFile. Alternatively, HIPAA-trained PMI staff upload the data into the participant's data folder in UAB ShareFile after the participant shares his or her data through the initial participant questionnaire. This data includes participants' health information such as their genetic information (i.e., genetic report, whole-genome or whole-exome sequencing data), medical history, clinical symptoms, imaging reports, and lab tests. Additionally, any data collected from the outcome survey that is given to participants after the consultation process is kept in UAB Sharefile.

The project management tool, Airtable^4^, is used to manage and track PMI cases as they pass through the consultation pipeline. No protected health information (PHI) is included in Airtable; only de-identified information is tracked using a unique PMI case identification number. De-identified information that is included in the Airtable case-tracking system includes genetic variants, Human Phenotype Ontology (HPO) terms, strictly limited participant information (year of birth, sex, state/country), and case status (e.g., intake, case analysis, report draft).

For both the initial participant questionnaire and outcome survey, PMI uses the Qualtrics Research Suite^5^. Through a business associate agreement with Qualtrics that covers HIPAA, PHI is collected and stored in Qualtrics. Responses to the questionnaire and survey are only accessible to authorized personnel included in the IRB study protocol.

### Case analysis process

Once the genetic and clinical information is gathered, a case is assigned to an analyst. The case analysis process has two stages. The first stage consists of gathering information from databases and published literature on what is known about the gene(s) and the encoded protein(s). Frequently used databases include GeneCards, NCBI Gene, gnomAD, ClinGen, UniProt, the Human Protein Atlas, and the Research Collaboratory for Structural Bioinformatics (RCSB) Protein Data Bank; a full list of resources is provided in Supplementary Table 1 (Berman et al., 2000; U.S. National Library of Medicine, 2004; Rehm et al., 2015; Uhlén et al., 2015; Karczewski et al., 2020; Safran et al., 2021; UniProt Consortium, 2021). These sources are consulted to understand the molecular and biological function of the gene harboring the variant, as well as its cellular and tissue localization, protein structure and important domains, related pathways, and the human disorders associated with the gene or variant. This stage provides the context and background information needed to hypothesize the disease mechanism, which is the second stage of the case analysis process. In the second stage, the specific genetic variant is scrutinized to determine its potential impact on protein function, i.e., biological implication. This is accomplished by determining the location of the variant with regard to important protein domains or regions and previously reported pathogenic variants, as well as obtaining functional studies or model organism data published in peer-reviewed literature. The four major categories of biological implications include loss of activity, toxic activity, underactivity, and overactivity (Might and Crouse, 2022). For each of these categories, there are several therapeutic options to consider, as well as queries that can be formulated for mediKanren.

### Using mediKanren for identifying drug candidates

In cases where up-regulating or down-regulating a gene could be beneficial, mediKanren helps identify drugs or compounds, as well as additional gene targets, that may accomplish the desired outcome. Results from mediKanren are then exported into a spreadsheet to allow the analysts to filter and sort to pull out relevant therapeutic options, with each result linked to an article in PubMed. Analysts read these articles to validate the result and determine the strength of evidence for the therapeutic(s) by considering the concentration of the drug used, the preclinical or clinical nature of the evidence, and the magnitude of the drug response. Top drug candidates are then prioritized based on accessibility, bioavailability, and safety.

### Evaluation of results

By design, return of results is not guided by any automatically calculated score from mediKanren. Instead, mediKanren is currently calibrated to return all (e.g., rare disease, drug) associations that are detectable with trusted data sets, and analysts manually review the results. Analysts predominantly use factors to evaluate the results that are not currently available in machine-processable data, including the meaning of text in returned articles, additional articles discovered by the analysts, current preclinical and clinical studies, and subject matter expertise on the case review team. Supplementary Table 1 resources also fall under the category of low machine accessibility, and thus resources that mediKanren currently does not know. Today, there is still a substantial amount of evidence required to produce a plausible research report that is hard to connect with automated methods.

In other domains, supervised labeling or benchmark data plays a major role. However, in our domain, no existing labeling or benchmark data sets rate efficacy for (e.g., rare disease, drug) pairs under consideration, due to two main factors. First, is the rareness of the diseases and their treatments. Second, is the significant analyst effort necessary to create each plausible labeled example (e.g., the research report). We have discussed publishing a corpus of cases which have risen to the level of a physician or research report (today over twenty participant cases), but at the project's current state of development, it is not yet clear how to publish such a corpus in a useful way.

### Returning results

In cases where an FDA-approved drug or well-studied nutraceutical is identified and predicted to positively impact the underlying disease mechanism, the results are vetted by the PMI clinical team. The proposed therapeutics that are deemed relatively safe by the medical directors are included in a research report that is shared with the participant's physician(s) of choice. This report includes a cover letter, participant background information, case analysis (i.e., gene/protein information and hypothesized variant impact), the mediKanren-identified therapeutic(s), and relevant publications.

### Case selection

A subset of cases was selected to highlight the utility of mediKanren and successful outcomes of the PMI program. These cases did not have a therapeutic option initially and were matched with at least one therapeutic through the case analysis process and subsequent use of mediKanren that went on to be trialed in a patient.

## Results

### “One-hop” query—Concepts connected by a single predicate return upregulators in predicted loss-of-function scenarios

In some cases, genetic variants lead to decreased activity of the protein (partial loss-of-function or hypomorph) or total lack of protein function (total loss-of-function or amorphic). In cases where the disease is dominant (one allele is affected), one allele remains functional. In these scenarios, identifying a strategy that can increase the activity of the remaining protein or gene may compensate for the lost function and rescue the harmful effects of the gene variant.

**Figure 1:**
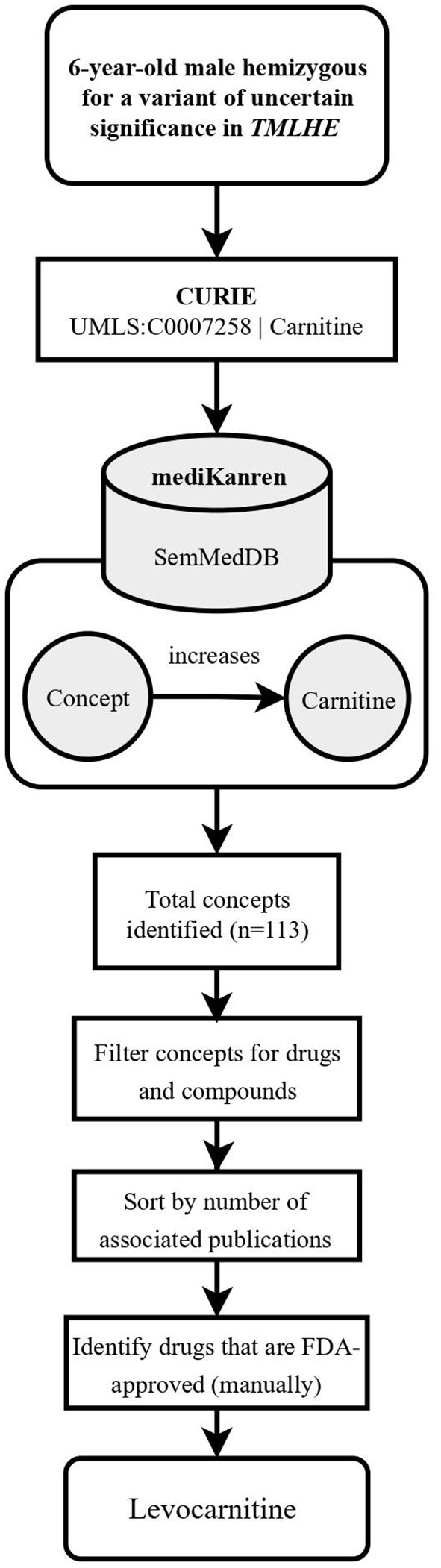
Flowchart illustrating the workflow of the *TMLHE* case using mediKanren. Based on the conclusions of the case analysis, the analyst used mediKanren to identify therapeutic strategies to compensate for the loss of trimethyllysine hydroxylase activity and decrease in carnitine biosynthesis. One hundred and thirteen concepts were returned by mediKanren using the SemMedDB KG that were predicted to increase carnitine, and of those, levocarnitine is FDA-approved and supporting literature was reviewed to confirm the result. *TMLHE*, Trimethyllysine Hydroxylase, Epsilon; CURIE, compact uniform resource identifier; UMLS, Unified Medical Language System; FDA, Food and Drug Administration.

One such case is a pediatric male participant hemizygous for an inherited missense variant in the gene *TMLHE*^6^, i.e., the protein epsilon-trimethyllysine hydroxylase, a key member of the carnitine biosynthesis pathway. Clinically, the participant experienced daily seizures, delayed speech development, and poor fine motor skills. During case analysis, an article was identified that reported another patient with a *TMLHE* variant that occurred near the PMI participant's variant (Nava et al., 2012). The functional analysis described in the article characterized the *TMLHE* variant as a loss-of-function, leading to an increase in trimethyllysine, the precursor of carnitine biosynthesis, and a slight decrease in carnitine levels (Nava et al., 2012). As a result, the PMI participant's variant was predicted to also result in a loss-of-function. Based on this analysis, the analyst used mediKanren to identify therapeutic strategies to compensate for the loss of trimethyllysine hydroxylase activity and decrease in carnitine biosynthesis. A mediKanren query for increasing carnitine revealed different forms of carnitine, including the FDA-approved dietary supplement levocarnitine (Figure 1). A research report outlining these findings, i.e., the case analysis and mediKanren results, was generated and sent to the participant's physician. Upon their review, the physician decided to move forward with a levocarnitine supplementation trial with their patient. Reports from the participant's family revealed that levocarnitine supplementation led to improvement of mobility. However, this information is anecdotal, and a full clinical trial is necessary to conclude that carnitine supplementation has a positive outcome for patients with this variant and others with similar impact on gene function.

Finding the *TMLHE* treatment involved identifying a drug or supplement that would increase metabolites within the carnitine biosynthesis pathway, thereby compensating for the loss of trimethyllysine hydroxylase function. In this instance, the desired result was returned as a single triple “levocarnitine increases carnitine.” When results are connected in a single triple, we refer to these as one-hop queries.

### “Two-hop” queries—Multiple concepts can be connected to identify additional results

Another common scenario encountered is where one-hop mediKanren queries do not lead to direct therapeutic outcomes, meaning there are no direct connections between the modulators and the gene. This could be the result of limited information known about the gene of interest or few potential therapeutics that target related cellular processes or pathways.

An example of this scenario was presented with two pediatric participants whose genetic sequencing revealed pathogenic missense variants in the gene *RHOBTB2*^7^ (Rho Related BTB Domain Containing 2). Clinically, these participants presented with seizures, severe global developmental delay and regression, paroxysmal movement disorder, and hypotonia. Missense variants in the RHOBTB2 protein have been linked to decreased dendritic development, resulting in decreased dendritic arborization (Straub et al., 2018). Case analysis revealed both participant variants are in the ubiquitin domain of the RHOBTB2 protein, which is important for the degradation of RHOBTB2. Functional studies in *Drosophila sp.*, along with case reports of affected individuals, have linked missense RHOBTB2 protein variants to decreased Rho GTPase protein degradation, increased protein accumulation in the central nervous system, and subsequent neuronal degradation, conferring a phenotype of developmental delay and epileptic encephalopathy (Belal et al., 2018; Straub et al., 2018).

**Figure 2:**
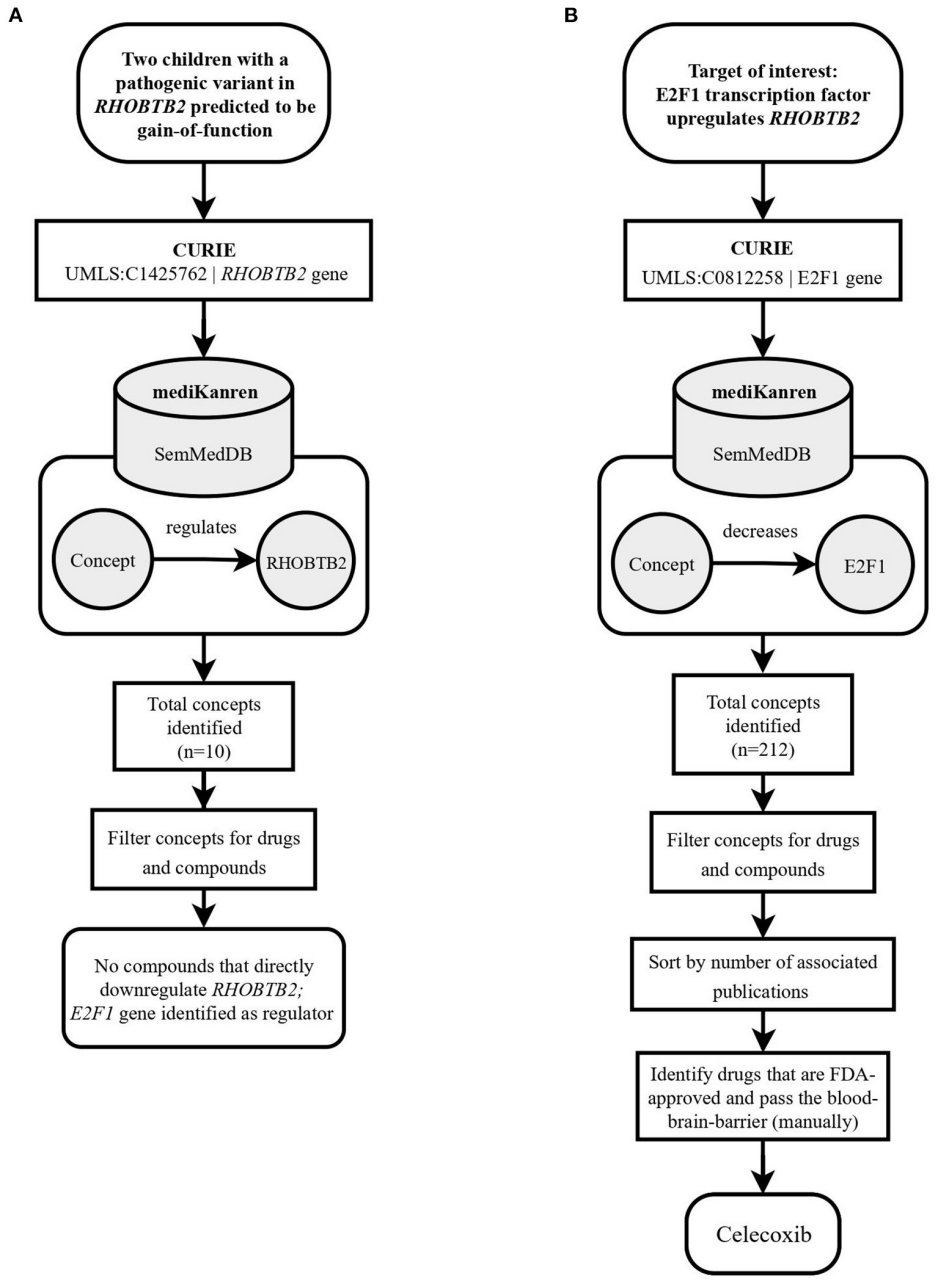
Flowchart illustrating the workflow of the *RHOBTB2* case using mediKanren. **(A)** Based on the conclusions of the case analysis, the analyst used mediKanren to identify therapeutic strategies that will regulate *RHOBTB2* gene expression. Ten concepts were returned by mediKanren using the SemMedDB KG that were predicted to regulate *RHOBTB2* expression, but none of the concepts were drugs or compounds. Therefore, a two-hop approach was taken with *E2F1*, a gene that was identified as a regulator of *RHOBTB2*. **(B)** A second query was run to look for downregulators of *E2F1*, which resulted in 212 concepts. After filtering for drugs, sorting by relevance, and manually reviewing for FDA-approved compounds that pass the blood-brain barrier, Celecoxib was identified. *RHOBTB2*, Rho Related BTB Domain Containing 2; CURIE, compact uniform resource identifier; UMLS, Unified Medical Language System; *E2F1*, E2F Transcription Factor 1.

The proposed therapeutic approach in these cases was to search for *RHOBTB2* gene downregulators. However, there was a lack of direct *RHOBTB2* inhibitors in the existing knowledge graphs in mediKanren at the time (Figure 2A). Therefore, another approach was taken. *RHOBTB2* itself has regulators, which provide additional targets for mediKanren queries. For example, the gene *E2F1*^8^, i.e., the protein E2F transcription factor 1, was identified by mediKanren as a positive regulator of *RHOBTB2* transcription (Freeman et al., 2008). As demonstrated in Figure 2B, a subsequent query of *E2F1* gene downregulators led to the identification of Celecoxib (Celebrex) (Dembo et al., 2005). This drug was selected because it was FDA-approved, well-tolerated in pediatric patients, and able to cross the blood-brain barrier. A research report for Celecoxib was generated and sent to each participant's physician. Upon their review, both physicians decided to move forward with a trial of Celecoxib in their patients.

Finding the *RHOBTB2* treatment pathway involved combining a drug-to-gene relation (Celecoxib downregulates *E2F1*) with a gene-to-gene relation (*E2F1* positively regulates *RHOBTB2*). Pathways of this shape are referred to as two-hop pathways, and queries to find two-hop pathways are known as two-hop queries.

### Long-tailed therapeutic hits—Query for when common therapeutics are not successful

**Figure 3:**
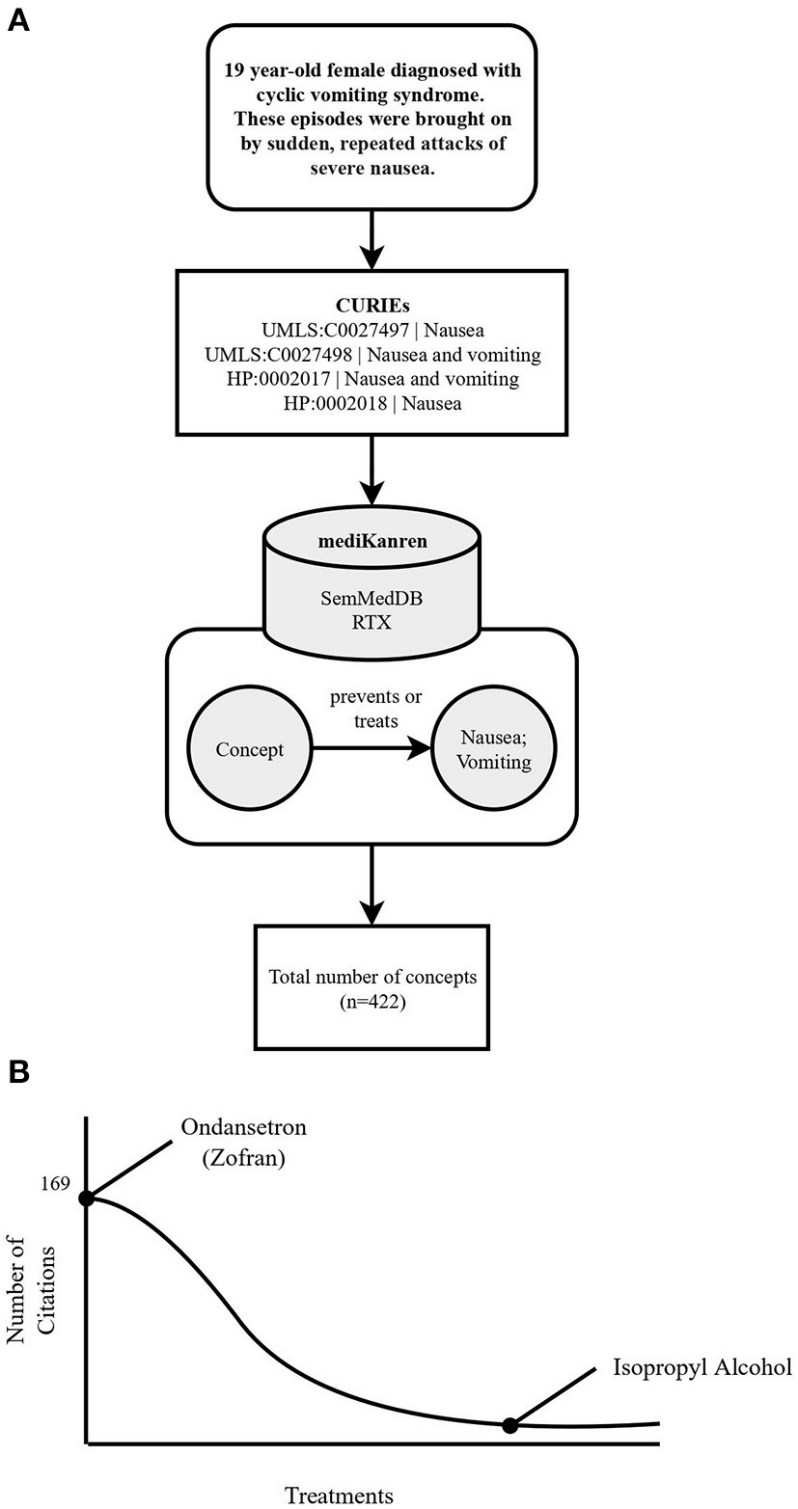
Flowchart illustrating the workflow of the cyclic vomiting syndrome case using mediKanren. **(A)** Over 422 concepts were returned by mediKanren using the SemMedDB and RTX KGs that were predicted to treat or prevent nausea and/or vomiting. **(B)** Ondansetron was the concept with the most publications, whereas isopropyl alcohol was located in the tail-end of the results with only three publications. CURIE, compact uniform resource identifier; UMLS, Unified Medical Language System; HP, human phenotype.

mediKanren is also invaluable when it comes to situations where a condition may have several standard of care treatments, yet a patient still does not achieve relief after trying multiple treatment options. An example of this scenario was a young adult female whose family reached out to PMI to find additional therapeutic options for their daughter's cyclic vomiting. Cyclic vomiting syndrome is a disorder that is generally debilitating, resulting in episodes of severe nausea and vomiting. These symptoms, nausea and vomiting, became the targets for mediKanren queries to help identify all therapeutics that could prevent these episodes. The results with the most support, i.e., the most publications, were common antiemetics such as Ondansetron (Zofran), which were already tried by the participant. However, in the long tail of the results, as shown in Figure 3, where there were only a few publications associated with each result, isopropyl alcohol was reported to prevent nausea (Smiler and Srock, 1998; Cotton et al., 2007; Beadle et al., 2016). Specifically, the cited publications referred to nasally inhaled isopropyl alcohol. This was a simple and safe strategy the young woman could try at the onset of her nausea to prevent vomiting episodes. Upon review by PMI medical directors, this therapy was shared with the participant's physician. This treatment was reported by the participant to provide relief from her nausea and vomiting symptoms. While some could characterize this finding as a “forgotten fact”, anecdotally, this strategy of preventing nausea is a well-known trick among nurses who need to combat nausea. However, when taken out of that context, it would be considered a “forgotten fact” as it appeared in the long tail of results for treating or preventing nausea and vomiting.

### Bioinformatics and AI pipelines to identify drug candidates for SARS-CoV-2

**Figure 4:**
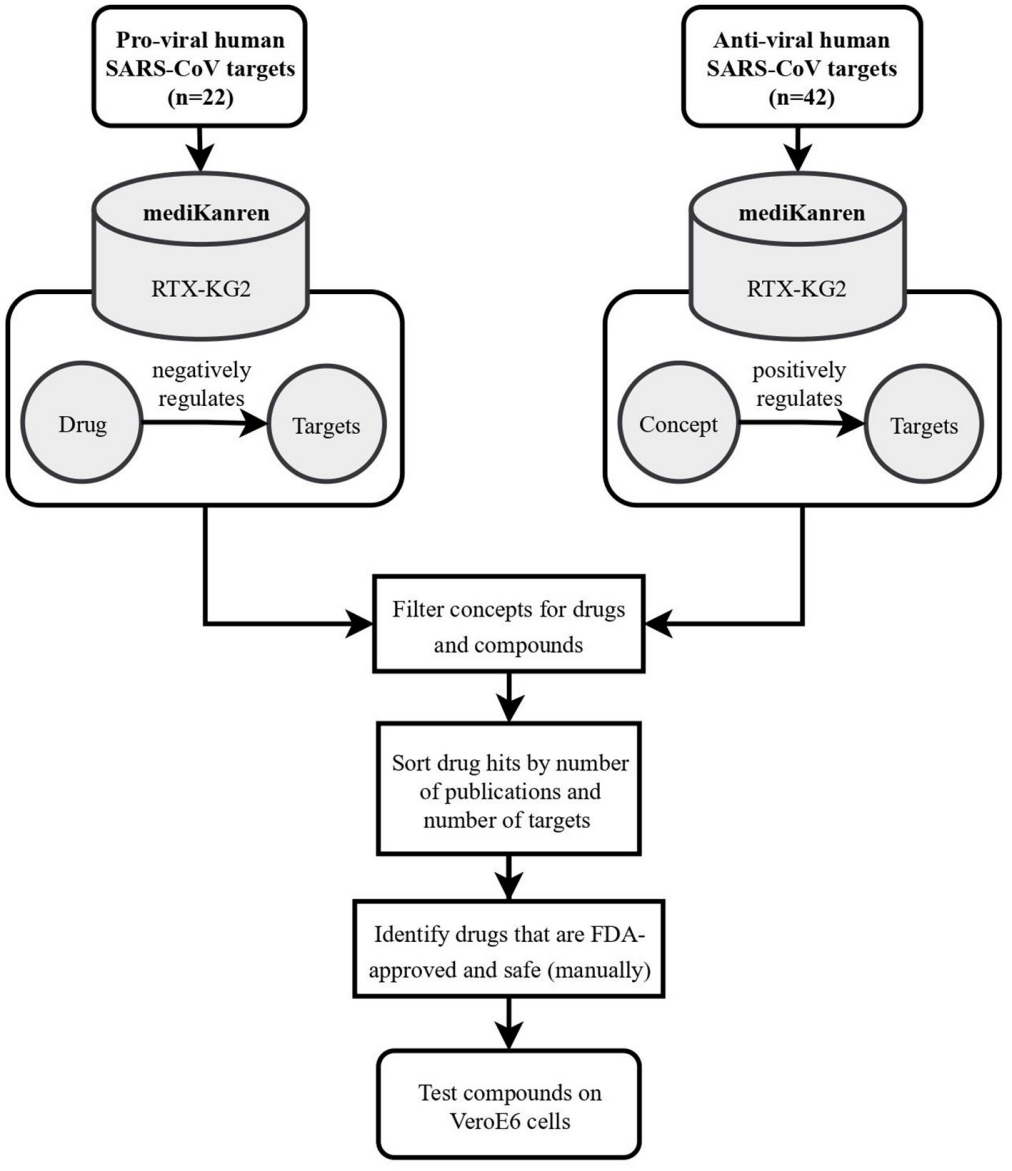
Flowchart illustrating the workflow of SARS-CoV-2 drug repurposing using mediKanren. Over 64 pro-viral and anti-viral targets were queried in mediKanren for negative and positive regulators, respectively. Over 1,300 drugs were returned by mediKanren using the RTX-KG2. These drugs were sorted by the number of publications and number of targets and manually reviewed to prioritize FDA-approved and safe drugs for testing in VeroE6 cells.

When the news of SARS-CoV-2 began to loom in early 2020, the PMI team applied the case analysis process for rare disorders to identify repurposable drugs for COVID-19. As reports detailing the predicted molecular mechanisms of infection and replication were released, a list of host and viral protein targets was prioritized and became the subjects of mediKanren queries (Figure 4). One target of interest, the endothelial cell surface protein *TMPRSS2*, was determined to be the receptor by which the virus entered lung tissue, and as a result, the strategy was to identify FDA-approved drugs that decrease *TMPRSS2* gene expression and therefore, decrease the ability of the virus to infect the host's lung tissue (Matsuyama et al., 2010). A mediKanren query for *TMPRSS2* gene regulators revealed that androgens induce *TMPRSS2* expression, thus a therapeutic strategy of androgen deprivation logically would decrease *TMPRSS2* expression. Accordingly, discussions regarding a prescription medication known as Degarelix were initiated between PMI and the United States Veterans Affairs Office of Research and Development. Degarelix is a prescription medicine used in the treatment of advanced prostate cancer that works as a luteinizing hormone-releasing hormone (LHRH) antagonist, thus leading to lowered testosterone levels in the body. As a result, the Hormonal Intervention for the Treatment in Veterans With COVID-19 Requiring Hospitalization (HITCH) (NCT04397718) was initiated to test this hypothesis (Nickols et al., 2021). Although the HITCH trial reported that androgen suppression through Degarelix treatment did not improve outcomes in a small cohort of men hospitalized with COVID-19, other trials found proxalutamide, an androgen receptor antagonist, was an effective treatment for non-hospitalized patients with COVID-19 (Cadegiani et al., 2021; McCoy et al., 2021; Nickols et al., 2022). Therefore, we cannot exclude the possibility that androgen suppression may reduce time to clinical remission and/or reduces the rate of hospitalization for patients with COVID-19.

In addition to identifying anti-androgen drugs as potential preventative repurposable drugs for COVID-19, over 1,300 other drugs were identified with proposed antiviral activity using mediKanren (de Wilde et al., 2015). These drugs were then ranked by the analysts according to the amount of evidence, safety and accessibility, and predicted interactions using molecular simulations. The top-ranked candidates were then validated in VeroE6 cells and successful candidates were further tested in a lung-organoid model of SARS-CoV-2 infection at Dr. Kevin Harrod's lab at UAB. One of the most promising mediKanren drug candidates was D-α-tocopherol polyethylene glycol succinate (TPGS), which was later found to interact with the SARS-CoV-2 RNA-dependent RNA polymerase and synergize with Remdesivir, thereby inhibiting SARS-CoV-2 replication in VeroE6 cells (Pacl et al., 2021).

## Discussion

Drug repurposing is a powerful approach to therapeutic discovery for rare and complex diseases. As biomedical discoveries continue to contribute vast datasets and knowledge, an opportunity emerges for AI to assist in translating this biomedical knowledge into a format that can be searched by clinicians and researchers to better understand these diseases and identify therapeutic strategies for patients. In this paper, we discussed how PMI developed the biomedical reasoning AI tool, mediKanren, and the complementary research consultation service that connects patients, researchers, and clinicians to the knowledge contained within mediKanren.

In two rare disease cases, *TMLHE* and *RHOBTB2*, analyses of genetic and health-related data and disease-related publications unraveled molecular mechanisms and pathways that provided therapeutic targets for mediKanren. In both cases, a novel therapeutic was discovered, shared with the participants' physicians, and was given to the participant. In a case of cyclic vomiting syndrome, mediKanren captured standard and lesser-known treatment options for alleviating nausea-related symptoms. One of these lesser-known treatment options greatly reduced and helped prevent the onset of nausea and vomiting episodes in a young female. The participant reported a significant improvement in her quality of life due to this mediKanren-identified treatment option that would have not been caught by a simple literature database search. In the complex infectious disease case of SARS-CoV-2, mediKanren unveiled the pathway of androgen-induced expression of *TMPRSS2*, which ultimately led to a clinical trial of androgen deprivation therapy for decreasing disease severity. Additionally, a search for drugs and compounds related to human SARS anti-viral and pro-viral targets uncovered over 1,300 drug repurposing candidates for screening in a cell model.

At the time of this paper, mediKanren has identified therapeutics that are FDA-approved and relatively safe in over twenty participant cases. For each of these cases, a report was shared with a physician, and it has been confirmed in over half of these cases that the physicians moved forward with the treatments. In addition to the cases mentioned in this paper, other rare disorders that have matched with therapeutic options include non-ketotic hyperglycinemia, *MAPK8IP3*-related disorder, *EGFR*-associated acanthosis nigricans, and *SETD1A*-related disorder. We are currently in the process of submitting IRBs for open-label clinical trials for other cases. Lastly, two mediKanren identified therapeutics have been tested in clinical trials: Degarelix for COVID-19 (NCT04397718) and ketamine for ADNP syndrome (NCT04388774)^9^ (Nickols et al., 2021).

### Obstacles and limitations

Unfortunately, most PMI cases do not result in a report to a physician for several reasons. The most common reasons include limited knowledge about a gene or variant, lack of mediKanren-identified therapeutics, or unsuitable therapeutic options (e.g., toxins, cancer treatments, treatments with significant side effect profiles). In these situations, more research is required before any treatment option is considered by a physician. Therefore, collaborations with researchers (basic and translational), foundations, and clinicians treating similar diseases are frequently sought out by PMI. The most fruitful and dynamic collaborations are with researchers who have models for specific genetic disorders and are willing to screen a limited number of drugs.

### Experience with the Semantic Medline Database

The Semantic Medline Database (SemMedDB) knowledge graph has been a cornerstone of the PMI consultation service. SemMedDB is a natural language processing (NLP) extraction of abstract-only text from PubMed. The NLP program that produces SemMedDB is SemRep (Kilicoglu et al., 2020). The RTX-KG2 knowledge graph incorporates SemMedDB, which is in turn consumed by mediKanren (Wood et al., 2021). PMI's case analysis process inspired the development of RTX-KG2 after Dr. Steve Ramsey noted how including the research article that supports an edge in the biomedical knowledge graph, especially the specific sentence excerpt on which the edge is based, is critical to enable analysts to maximally leverage human judgment in the interpretation of mediKanren results. Accordingly, RTX-KG2 provides literature provenance information wherever it is available, such as for edges from SemMedDB, Jensen Lab Diseases, and RepoDB. Feedback from PMI based on experiences using RTX-KG2 within mediKanren led to multiple improvements for RTX-KG2 including improvements to semantic type annotations, the documentation of constituent sources, and additions of knowledge sources, such as DGIdb (Wood et al., 2021).

SemMedDB also has shortcomings that reveal research opportunities with high impact, both scientific and humanitarian. For instance, the predicates in SemMed have varied widely in their applicability to PMI's work. Predicates “inhibits” (i.e., biolink:negatively_regulates) and “stimulates” (i.e., biolink:positively_regulates) have been indispensable, while vague predicates such as “associated_with” (i.e., biolink:related_to) have been much less helpful. Additionally, each structured datum in SemMedDB includes the PubMed text sentence with which it matched; the matched text has proved helpful in allowing users to manually throw out low confidence matches before investing time in reading the abstract or article. However, even when full PubMed article text has open access copyright, and is therefore likely to contain useful matches within the text, SemMedDB/SemRep is limited to only reading the abstract.

The authors of SemMedDB/SemRep recently used the BioCreative V benchmark to compare their system with two newer (2016) systems and concluded that the two newer systems performed better on F1 score and much better on recall (Peng et al., 2016; Xu et al., 2016; Kilicoglu et al., 2020). High recall is necessary when the goal is to investigate all possibilities, as is the case for participants who match to only a few or no drugs. Therefore, integration with newer NLP techniques could make a substantial improvement. Lastly, NLP systems cannot access articles behind copyright paywalls. These articles may contain information on life-saving therapeutic options, but unless the research findings become available to the public and accessible by NLP systems, NLP systems cannot help in making the findings accessible.

### Future directions

With the development of mediKanren and the research consultation service, PMI has had a significant impact on many individuals, patient communities, as well as the field of translational science. To scale up this process and help reach more individuals, a few key elements must be addressed.

One challenge to address is keeping up with the large number of patient referrals to PMI. A current strategy is the creation of formal training modules that will teach UAB undergraduates how to analyze genetic information, assess the variant impact, formulate a molecular hypothesis, and use mediKanren to identify potential therapeutics. Through remote online learning, additional undergraduates at participating institutions can also be trained as precision medicine analysts. By having students go through an onboarding process, they will feel more confident in applying their knowledge and skills to participant cases. Additionally, they will gain experience with each case they take on, thus gradually growing their knowledge and developing their critical thinking skills. This will allow for more students from any background and previous training, or lack thereof, to be a part of the process, and therefore, gain relevant research and medical training while expanding the number of active cases in the program at any given time.

There are also various improvements that can be made to mediKanren, to not only improve the accuracy of the results but also expand access to new features such as knowledge graphs and potential queries that can be run. Lastly, progress is underway to have a web interface for the NCATS Translator project's reasoners, including mediKanren. We hope this effort will ultimately make mediKanren easily usable by all interested parties. PMI plays an active role as a source of subject matter experts (SMEs) for the NCATS Biomedical Data Translator program, providing insights into what features and knowledge graphs would help accelerate and improve the research consultation process and better serve researchers and clinicians.

## Data availability statement

The original contributions presented in the study are included in the article/Supplementary material, further inquiries can be directed to the corresponding author/s.

## Ethics statement

The studies involving human participants were reviewed and approved by the University of Alabama at Birmingham Institutional Review Board. Written informed consent to participate in this study was provided by the participants' legal guardian/next of kin.

## Author contributions

MM, AC, and WB contributed to the conception and design of mediKanren and the PMI research consultation service. WB designed and implemented the mediKanren graphical user interface. GR designed and implemented the graph database engine and reasoning back-end of mediKanren, made improvements to the mediKanren graphical user interface, and wrote the mediKanren section of the manuscript. AF, CC, JH, WB, GR, and AC wrote the sections of the manuscript. AF and CC wrote the first draft of the manuscript. WB, JH, GR, MP, KH, TT-N, MZ, JO, and NA made technical contributions to mediKanren. SR contributed essential knowledge graphs for mediKanren. NA worked with WB on queries for COVID-19 drug repurposing. MZ wrote advanced mediKanren queries to explore advanced precision medicine queries, with guidance from MP, TT-N, GR, WB, and NA. UAB Precision Medicine Institute performed the case analysis and consultation process of the research consultation service. All authors contributed to manuscript revision, read, and approved the submitted version.

## Funding

Support for this work was provided by the National Center for Advancing Translational Sciences, National Institutes of Health, through the Biomedical Data Translator Program, awards OT2TR003435, OT2TR002517, and OT2TR003428. Any opinions expressed in this document are those of the Translator community at large and do not necessarily reflect the views of NCATS, individual Translator team members, or affiliated organizations and institutions. Support for MZ was provided by the Harvard College Research Program (HCRP) through the Harvard College Office of Undergraduate Research and Fellowships.

## UAB Precision Medicine Institute

Stephen Barnes, William E. Byrd, Mei-Jan Chen, Andrew B. Crouse, Camerron M. Crowder, Mary E. Crumbley, Madeline Eckenrode, Crayton A. Fargason, Jr., Nathaniel Fehrmann, Aleksandra Foksinska, Kaiwen He, Forest Huls, Matthew Jarrell, Lindsay Jenkins, Meg McCalley, Matthew Might, Tamsyn Osborn, Michael J. Patton, Elizabeth Pollard, Gregory Rosenblatt, Sienna Rucka, Nicholas T. Southern, Thi K. Tran-Nguyen, Jillian Tinglin, and Jordan H. Whitlock.

## Conflict of interest

Author JH is employed by Groovescale. The remaining authors declare that the research was conducted in the absence of any commercial or financial relationships that could be construed as a potential conflict of interest.

## Publisher's note

All claims expressed in this article are solely those of the authors and do not necessarily represent those of their affiliated organizations, or those of the publisher, the editors and the reviewers. Any product that may be evaluated in this article, or claim that may be made by its manufacturer, is not guaranteed or endorsed by the publisher.

## Footnotes

^1^Global Genes (2021). *RARE Disease Facts. Global Genes*. Available online at: https://globalgenes.org/rare-disease-facts/ (accessed March 25, 2022).

^2^W3C (2010). *CURIE Syntax 1.0*. Available online at: https://www.w3.org/TR/curie/ (accessed March 27, 2022).

^3^Citrix ShareFile. Available online at: https://www.citrix.com/lp/sharefile/ (accessed March 23, 2022).

^4^Available online at: https://www.airtable.com/ (accessed March 18, 2022).

^5^Qualtrics (2005). *Qualtrics*. Available online at: https://www.qualtrics.com (accessed March 18, 2022).

^6^NCBI TMLHE trimethyllysine hydroxylase, epsilon [*Homo sapiens* (human)]. Gene. Available at: https://www.ncbi.nlm.nih.gov/gene/55217 (accessed March 18, 2022).

^7^NCBI RHOBTB2 Rho related BTB domain containing 2 [*Homo sapiens* (human)]. Gene. Available at: https://www.ncbi.nlm.nih.gov/gene/23221 (accessed March 17, 2022).

^8^NCBI E2F transcription factor 1 [*Homo sapiens* (human)]. Gene. Available online at: https://www.ncbi.nlm.nih.gov/gene/1869 (accessed March 18, 2022).

^9^NIH National Library of Medicine ClinicalTrials.gov. *Low-Dose Ketamine in Children With ADNP Syndrome*. Available online at: https://clinicaltrials.gov/ct2/show/NCT04388774 (accessed March 18, 2022).
