# Supplementary material for: The precision medicine process for treating rare disease using the artificial intelligence tool mediKanren
Source: Front Artif Intell. 2022 Sep 30;5:910216. doi: 10.3389/frai.2022.910216 (PMC9562701; doi:10.3389/frai.2022.910216)
Supplement: Supplementary file 1 [file Table_1.DOCX]

**SUPPLEMENTAL TABLES AND FIGURES**

**Supplemental Table 1.** External resources frequently used by PMI analysts to gather information about specific genetic disorders or diseases.

| **Name** | **Website** | **Brief Description** |
| --- | --- | --- |
| PubMed | <https://www.ncbi.nlm.nih.gov/pubmed/> | Search engine for an extensive collection of biomedical literature |
| ClinicalTrials.gov | <https://clinicaltrials.gov/> | Database of national and international clinical trials |
| SFARI Gene | <https://gene.sfari.org/> | Database of genes/chromosomal abnormalities associated with autism |
| GeneCards | <https://www.genecards.org/> | Database of extensive gene-associated information |
| VarSome | <https://varsome.com/> | Library of variant and gene annotation |
| gnomAD | <https://gnomad.broadinstitute.org/> | Summarizes large-scale exome and genome sequencing data |
| UCSC Genome Browser | <https://genome.ucsc.edu/> | Visualization tool for genomic data |
| SnapGene | <https://www.snapgene.com/> | Visualization tool for annotated sequence files |
| NCBI gene | <https://www.ncbi.nlm.nih.gov/gene> | Portal for transcript-specific knowledge for genomic data |
| UniProt | <https://www.uniprot.org/> | Database of protein sequence/functional information |
| The Human Protein Atlas | <https://www.proteinatlas.org/> | Extensive collection of human protein expression and localization data |
| RCSB Protein Data Bank (PDB) | <https://www.rcsb.org/> | Information about protein structure, ligands, etc. |
